# Soil coring at multiple field environments can directly quantify variation in deep root traits to select wheat genotypes for breeding

**DOI:** 10.1093/jxb/eru250

**Published:** 2014-06-24

**Authors:** A. P. Wasson, G. J. Rebetzke, J. A. Kirkegaard, J. Christopher, R. A. Richards, M. Watt

**Affiliations:** ^1^CSIRO Plant Industry, GPO Box 1600, Canberra, ACT 2601,Australia; ^2^Queensland Alliance for Agricultural and Food Innovation, University of Queensland, Leslie Research Centre, PO Box 2282, Toowoomba Queensland 4350, Australia

**Keywords:** Deep roots, root penetration rate, physiological selection, soil coring, field phenotyping, wheat breeding.

## Abstract

We aim to incorporate deep root traits into future wheat varieties to increase access to stored soil water during grain development, which is twice as valuable for yield as water captured at younger stages. Most root phenotyping efforts have been indirect studies in the laboratory, at young plant stages, or using indirect shoot measures. Here, soil coring to 2 m depth was used across three field environments to directly phenotype deep root traits on grain development (depth, descent rate, density, length, and distribution). Shoot phenotypes at coring included canopy temperature depression, chlorophyll reflectance, and green leaf scoring, with developmental stage, biomass, and yield. Current varieties, and genotypes with breeding histories and plant architectures expected to promote deep roots, were used to maximize identification of variation due to genetics. Variation was observed for deep root traits (e.g. 111.4–178.5cm (60%) for depth; 0.09–0.22cm/°C day (144%) for descent rate) using soil coring in the field environments. There was significant variation for root traits between sites, and variation in the relative performance of genotypes between sites. However, genotypes were identified that performed consistently well or poorly at both sites. Furthermore, high-performing genotypes were statistically superior in root traits than low-performing genotypes or commercial varieties. There was a weak but significant negative correlation between green leaf score (–0.5), CTD (0.45), and rooting depth and a positive correlation for chlorophyll reflectance (0.32). Shoot phenotypes did not predict other root traits. This study suggests that field coring can directly identify variation in deep root traits to speed up selection of genotypes for breeding programmes.

## Summary

Variation in deep root traits among wheat genotypes were identified in the field using high-throughput soil coring. Some weakly significant relationships were found between deep root traits and above-ground surrogates.

## Introduction

Trends in global wheat yields show that the rate of increase per year is too slow to meet population increases and demands projected for the twenty-first century (Fischer and Edmeades, 2010; Hall and Richards, 2013). Currently, wheat yield gains of 0.9% per annum are achieved through conventional breeding methodologies, whereby breeding organizations select the highest yielding genotypes among thousands tested with the quality characteristics and appropriate disease resistance. Research organizations aim to increase the speed of yield gain by identifying beneficial traits, and providing genotypes with those traits to breeders, so that these traits can be incorporated into new varieties to increase yields faster than selecting for yield alone (Richards *et al.*, 2010). Such trait-based approaches underpinned the Green Revolution when wheats with a shorter plant height were supplied to Indian and Pakistani farmers (Byerlee and Moya, 1993).

This paper focuses on deeper root systems as a trait to increase wheat yield. A number of papers have used modelling to demonstrate that deeper root systems are likely to confer a yield advantage in environments where water is available deeper in the soil profile at the time of grain filling (e.g. Asseng *et al.*, 1998; Manschadi *et al.*, 2006; Hammer *et al.*, 2009; Lilley *et al.*, 2011). Kirkegaard *et al.* (2007) demonstrated with direct root and soil water measurements in the field, that increases in root system depth (up to 30cm) could capture an extra 10mm of deep soil water at the time of grain development (between flowering and maturity) resulting in an extra 0.5 tonnes of grain per hectare. This deep, late-season water is valuable because it contributes directly to the allocation of carbohydrate to grain. Lopes and Reynolds (2010) found that amongst isomorphic wheat sister lines those with increased rooting depth had superior adaptation to drought where water was available at depth. Deeper roots can contribute disproportionately to water uptake and yield, with Gregory *et al.* (1978) showing that winter wheat roots below 100cm in depth contained 3% of the total root weight but were responsible for supplying 20% of the transpired water. Deeper rooting is also a useful trait to stabilize yields across seasons: deep water is protected from evaporation and is partly predictable because it can be measured and sometimes managed by the farmer before the crop is sown (Wasson *et al.*, 2012; Kirkegaard and Hunt, 2010).

Variation for root architecture amongst crops is comprehensively reviewed in O’Toole and Bland (1987). A quantitative trait loci (QTL) linked to root growth angle in rice, DRO1, has been found to be associated with deeper root growth in the field and its introgression into a shallow-rooted rice variety was associated with drought stress avoidance (Uga *et al.*, 2013). Molecular markers for root traits in rice were also associated with greater root length in the field in the work of Steele *et al.* (2006). There are complex relationships between root length, rooting depth, water uptake, and growth stage. In potato, the root length at each soil depth was correlated with water uptake at those depths only early in the season, with roots deeper in the profile contributing a disproportionate amount of the total crop water requirement irrespective of the status of roots in shallower layers later in the season (Stalham and Allen, 2004).

Considerable effort has gone into identifying variation among species and genotypes for root length and/or angle with the aim of developing deeper-rooted varieties. Assessment for increased root depth is largely undertaken in controlled environments; for example, root boxes (Hurd, 1974), wax-penetration systems (Botwright Acuña *et al.*, 2007), baskets (Oyanagi *et al.*, 1993), and germination papers (Watt *et al.*, 2013). Screens in controlled environments offer the advantage of examining a large number of genotypes with reduced environmental variation. However, it is unclear if these controlled environment screens, generally conducted on seedling root systems, translate to deeper roots in the field at the time of grain development, when deep water capture is expected to contribute significantly to yield. Few studies have compared the controlled environment screen to rooting depth in mature field-grown crops (Watt *et al.*, 2013). Indeed, to date no varieties are known to have been released based on selection for deeper roots in controlled environments. Deep root traits are (i) expressed in mature wheat plants, and (ii) are heavily influenced by edaphic factors, making it difficult to study them in a meaningful way in the laboratory. Therefore, in contrast to the usual approaches, there may be an advantage to first selecting superior genotypes in the field to speed up the identification of the best germplasm for use in breeding programmes (Rich and Watt, 2013; Wasson *et al.*, 2012).

In this study we investigated genetic variation for deep root phenotypes at maturity in the field because this is the critical time for trait expression to provide a yield benefit. The root systems were measured directly using soil coring. The aims of the study were: (i) to assess genetic variation for root traits at the time of grain development in a population selected for physiological characteristics thought to contribute to root growth using a rapid soil coring technique and a hill plot sowing configuration; and (ii) to assess the association between proposed indirect measures of root performance (canopy temperature, stay-green and chlorophyll reflectance) and destructively measured root traits. The purpose of the study was to determine if deep-rooted wheats can be identified by direct coring in field environments to speed up their incorporation into breeding programmes.

## Materials and methods

We applied two approaches to increase our chances of identifying deep rooted genotypes: (i) we used diverse germplasm that included ‘physiological types’ thought to increase rooting depth; and (ii) we used a novel planting configuration, called ‘hill’ plots, to assess large numbers of genotypes in a small space, promote root growth downward and create a uniform environment around the wheats.

### Germplasm: physiological types and commercial varieties

The wheats sown in the experiment were ‘spring wheats’, which do not require a period of cold to flower (vernalization); however, in Australia they are typically sown in autumn and harvested in the summer. The wheat germplasm represented a diverse set of experimental breeding lines (genotypes) referred to as ‘physiological types’, Australian commercial varieties, and released Indian varieties (Table 1). Triticales, which are crosses of wheat with rye species for improved environmental tolerance, were also included because they exhibit vigorous early growth and extensive root systems. The wheat ‘physiological types’ were (i) near-isogenic lines (NILs), being nearly identical genotypes with and without the tiller-inhibiting, *tin* gene that reduces the number of wheat shoots, and can be associated with increased root growth (Richards *et al.*, 2007); (ii) vegetatively vigorous material derived from the genotype Vigour 18, conferring rapid shoot growth and early root growth in unploughed soil and sandy soil (Spielmeyer *et al.*, 2007; Watt *et al.*, 2005; Liao *et al.*, 2006; Pang *et al.*, 2014); (iii) synthetic wheats selected in drying profiles in the field (Trethowan *et al.*, 2004); (iv) genotypes containing alternative dwarfing genes, which may expend energy saved in reduced shoot development in increased root development (Rebetzke *et al.*, 2012); and (v) progeny from crosses between Seri and Babax, which are globally well-adapted recombinant inbred lines (RILs) and a potential source of deep roots based on leaf greenness and cool canopies in droughted conditions (Olivares-Villegas *et al.*, 2007). A small number of wheat varieties from India, with contrasting adaptation to irrigated and rainfed conditions, were included, alongside a diverse, random selection of wheats including varieties released for dry conditions in Australia.

**Table 1.** Germplasm included in the trial

Physiological type	Description	Adaptation	Pedigree	Germplasm
Alternative dwarfing background	Material with alternative dwarfing genes (i.e. differing to the *Rht- B1b* or *Rht-D1b*). Reduced shoot growth may increase the partitioning of carbohydrates to the root system.	Slow and quick, spring- maturing wheats of dwarf, semi-dwarf and tall stature.	All lines are back-cross derivatives of Australian commercial varieties.	M808S
Alternative dwarfing genes				LAN1^a^, LAN8^b^ *Leeton only:* LAN2 *Bethungra only:* LAN4
Height pair	Near isogenic pair from alternative dwarfing gene cross, differing for height.	Spring maturing wheats.	Backcrossed from M808S and LAN13, which carries an alternative dwarfing gene.	*Leeton only:* ML80Tall, ML45Short
Indian rainfed	Released varieties for rainfed wheat production in India.	Spring maturing wheats.	Diverse genetic sources.	C306^a^, Dhawardry^a^
Indian irrigated	Released varieties for irrigated wheat production in India.	Spring maturing wheats.	Diverse genetic sources.	DBW14^a^, DBW16, DBW17, Raj3765
Spring commercials	Germplasm representative of wheats grown commercially in Australia.	Spring-maturing wheats of semi-dwarf stature.	Diverse genetic sources.	Yenda^a^ *Leeton only:* H45, Bolac, Diamondbird^b^, Hartog, Janz, Hartog *Bethungra only:* Drysdale
Synthetic	Synthetic wheat varieties identified in controlled environment screens as having rapid seedling root growth.	Spring-maturing wheats of tall or semi-dwarf stature.	Diverse primary and secondary derivatives in CIMMYT-based backgrounds.	30374, 33404^a,b^, Syn29589
Tin–	Near isogenic pairs of spring wheat varieties with and without the tiller-inhibiting *tin* gene. Reduced shoot growth may increase the partitioning of carbohydrates to roots.	Slow and quick, spring- maturing wheats of semi- dwarf stature.	All lines are top- or back-cross derivatives of Australian commercial varieties.	6336N^a^
Tin+				6336P2^a^
Triticale	Triticales were included for their vigorous early growth and extensive root systems.	Spring-maturing triticales of tall or semi-dwarf stature.	Diverse genetic sources.	*Leeton only:* Abacus, Bogong, Currency, Speedee
Vigour	The Vigour 18 germplasm was identified in a controlled environment screen for rapid seedling root growth. It has subsequently been used as a donor in crosses into commercial spring wheat backgrounds.	Spring-maturing wheats of tall or semi-dwarf stature.	All lines are top-cross derivatives of Australian commercial varieties.	38-19, CV100, CV109, CV445^b^, Vigour18^b^, JV22 *Bethungra only:* FV25
Other				NIL3-14 *Leeton only:* BC1-442, 92-11(2), BC1-431, SB20, Babax

^a^ Also included at Kingsthorpe in 2011.

^b^ Also measured in 2010 and 2009.

### Field environments—locations and climates

**Fig. 1.**
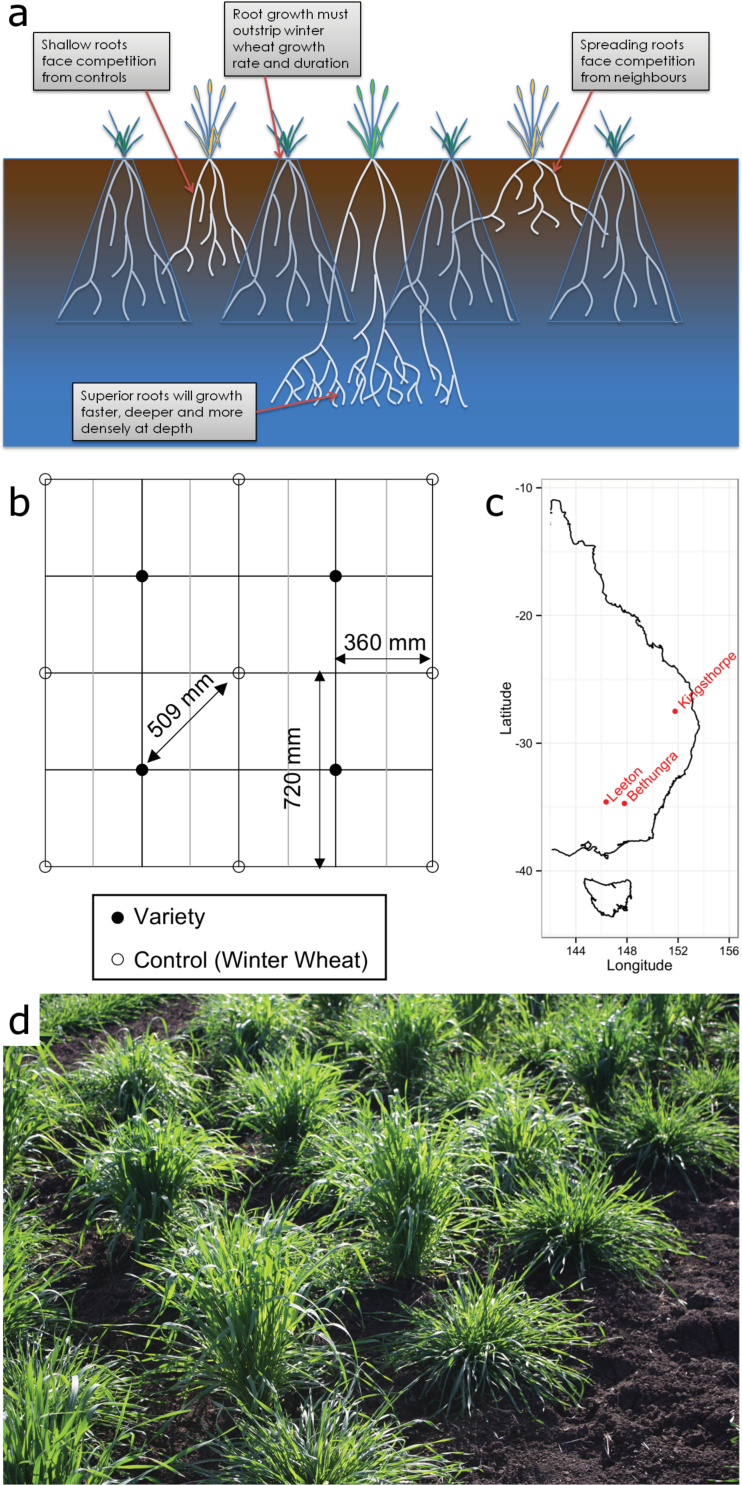
Trial design rationale. (a) A cartoon of hill plot concept. The lines being evaluated were to be grown in hill plots, and hypothetical variation in root distribution is shown. Each line was to be surrounded by control hill plots of uniform winter wheat. The winter wheat, sown in a spring wheat sowing window, would continue vegetative growth as the lines matured. This would force the lines to compete with controls with deep roots and continuing water uptake. The rationale was that this would provide a competitive advantage to lines with deeper and denser root systems, whereas lines with shallow roots or spreading roots would have to compete with the controls, thus linking desirable root traits with performance. (b) Shows the grid planting pattern for hill plots used in 2011. Furrows were pulled with a 180mm spacing (vertical black and grey lines). Hill plots were sown in every second furrow, with sowing alternating between lines and controls. Hill plots were sown with a 720mm spacing in the furrow, with the sowings staggered by 360mm between control and line furrows, so that each line was surrounded diagonally by four controls at a distance of 509mm. (c) Shows the location of the trial sites on the east coast of Australia. (d) Shows hill plots sown in Queensland in 2011. The wheats are in a vegetative growth stage. The shorter, prostrate hill plots are winter wheat controls. The taller hill plots are lines.

The three field environments were (i) Leeton, NSW (34° 36′S, 146° 22′E, 138 m elevation) on a grey vertosol soil (soil types are described in Isbell (2002)); (ii) Bethungra, NSW (34°43′S, 147°48′E, 310 m elevation) on a red kandosol soil; and (iii) Kingsthorpe, QLD (27°30′S,151°46′E, 440 m elevation) on a black vertosol soil (Table 2 and Fig. 1c). Soil and rainfall are detailed in Table 2. There were no known chemical or physical constraints to root growth at these sites to a depth of at least 2 m. The experiments were sown in 2011 in late May in Bethungra and Leeton and in June in Kingsthorpe. Limited measurements of root systems were performed on other trials performed at these sites in 2009 and 2010.

**Table 2.** Trial sites with characteristics and management details

Site	Location	Soil type^a^	Rotation	Rainfall^b^		Soil characteristics^c^		
Depth (cm)	Bulk density (g/cc)	Wheat PAWC (mm)
Bethungra, NSW	34°43′S, 147°48′E	Red Kandosol	Canola	Average annual	609	0–15	1.532	25.4
						15–30	1.614	25.1
						30–60	1.633	47.4
						60–90	1.769	27.6
				2011 (in-season)	770	90–120	1.645	44.7
						120–150	1.669	24.9
						150–180	1.655	6.9
						*Total PAWC mm*		*202*
Leeton, NSW	34° 36′S, 146° 22′E	Grey Vertosol	Pasture/ Grazing	Average annual	401	0–15	1.473	24.6
						15–30	1.438	19.2
				2009 (in-season)	289	30–60	1.431	40.8
						60–90	1.499	32.7
				2010 (in-season)	548	90–120	1.577	29.1
						120–150	1.59	26.7
				2011 (in-season)	591	150–180	1.489	17.7
						*Total PAWC mm*		*190.8*
Kingsthorpe, QLD	27°30′S, 151°46′E	Black Vertosol	…	Average Annual	632	0–15	0.9	33
						15–30	1.01	28.5
						30–60	1.02	54
						60–90	1	54
						90–120	1.06	36
						120–150	1.14	39
						150–180	1.23	0
						*Total PAWC mm*		*244.5*

^a^ Isbell (2002).

^b^ Based on Australian Bureau of Meteorology weather station records (www.bom.gov.au).

^c^ Based on nearest APsoil sampling location to site: Bethungra, No. 180, Leeton No. 174, Kingsthorpe No. 30, Gatton No. 37 (http://www.apsim.info/Products/APSoil.aspx).

### Experiments: planting configuration and management

The ‘hill’ sowing configuration was intended to maximize the contribution deep roots would make to plant performance. The concept is illustrated as a cartoon in (Fig. 1a), showing the hypothetical variation in rooting distribution and the theoretical consequences. ‘Hill plots’ are a sowing technique used in cereal breeding to assess harvest index and yield whilst minimizing sowing area (Bonnett and Bever, 1947; Frey, 1965; O’Brien *et al.*, 1979; Blum *et al.*, 1982; Tragoonrung *et al.*, 1990). Our strategy was to create a grid of control hill plots between which our genotypes for evaluation would be grown and assessed (Fig. 1b). The strategy was intended to provide: (a) uniform neighbour competition (Rebetzke *et al.*, 2014), (b) strong competition for available soil water in the subsoil using longer-maturing winter wheat, (c) high root system density (for soil sampling), and (d) greater soil uniformity in a small planting area. We are not aware of any studies contrasting root development in hill plots and standard row plots. However, we anticipate that the higher sowing density results in a higher root system density but that the comparative expression of root traits between genotypes will remain the same.

Germplasm were sown as hill plots in a configuration represented in Fig. 1b, d. A tractor-drawn seeding machine was used to generate 10 rows and incorporate fertiliser (Starter 15®, Nitrogen:Phosphorus:Potassiums:Sulphate S: 15:13:0:11 at 110kg ha^–1^), but seeds were not sown with the machine. Instead hill plots were sown into the rows by hand, by pouring ~30 seeds down a 40mm diameter PVC tube and covering with ~30mm of soil. The use of the PVC tube allows the operator to sow the seeds while standing and deposits the seeds in a small clump. Each hill plot was spaced within the row, with a spacing of 720mm (1.0 m at Gatton). The rows were spaced 180mm apart in Leeton and Bethungra, NSW and 250mm apart in Kingsthorpe, Queensland, and at a depth of 20–40mm. Every experimental genotype was surrounded on four sides by a common winter wheat. The winter wheat was chosen to provide a common environment around each experimental genotype, and because its later maturity would ensure a longer vegetative stage and thus deeper rooting to provide ongoing above- and below-ground competition throughout the reproductive stage.

Hill plots were maintained free of weeds by application of recommended pre- and post- emergent herbicides and where necessary by hand weeding. Prophylactic fungicide spray was carried out to minimize damage by leaf diseases such as yellow rust.

### Root measurements

**Fig. 2.**
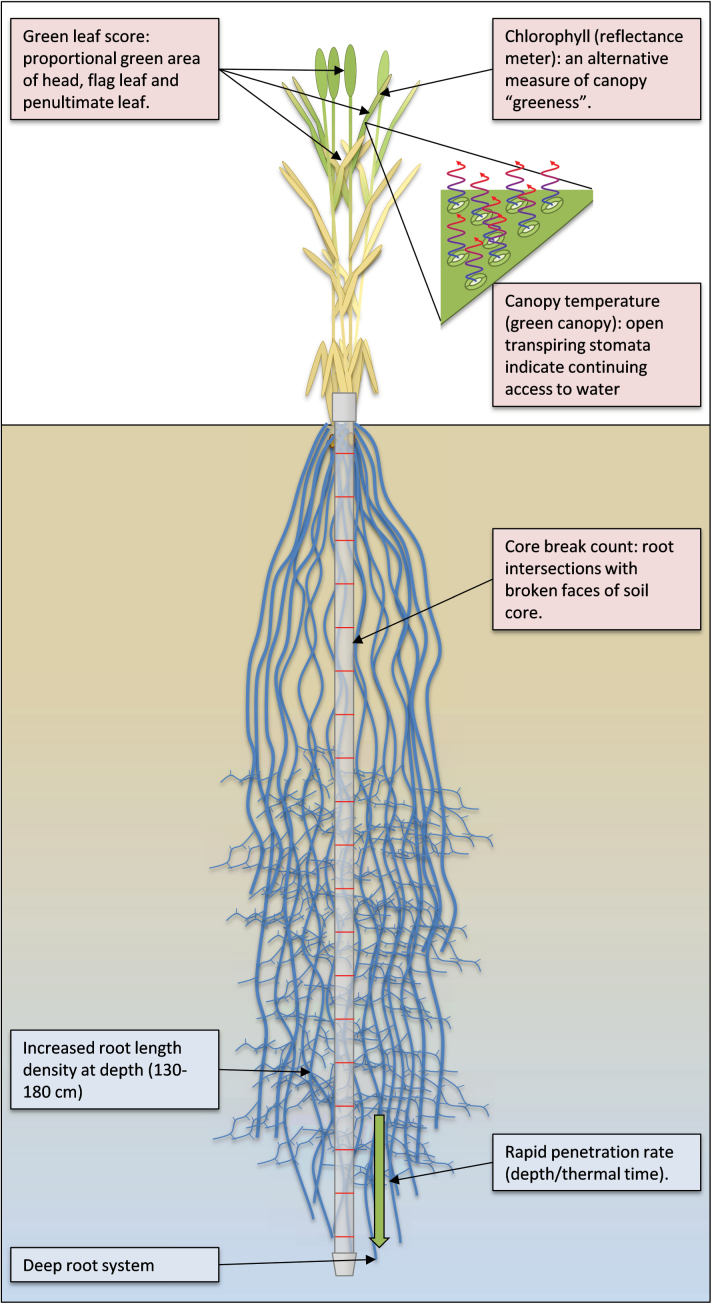
Cartoon of traits and measurements in Hill Plots. Traits of interest (blue tinted boxes) are (1) a deep root system generated by (2) rapid root penetration rate and (3) increased root density at depth (in the 130–180cm layer). Penetration rate is conceived as depth per unit of thermal time, not as root tip development rate; thus it may integrate different physiological traits such as the ability to grow through hard soil or to find and exploit cracks and pores. Measurements performed (green tinted boxes) included direct measurements of roots, namely (1) soil coring with core break counting of the root intersections with the broken faces of soil core (every 10cm), and indirect measures potentially related to root performance including (2) green leaf scoring after flowering as a measure of the ‘stay-green’ trait, (3) chlorophyll meter measurements as an alternative measure of stay-green, and (4) canopy temperature measurements as a proxy for open transpiring stomata indicating continued access to water.

Soil coring was used to directly phenotype the roots (Fig. 2). Root traits measured were (a) a deep root system, (b) rapid root penetration rate (depth at maturity/thermal time to flowering (anthesis)), and (c) increased root length density at depth. To manage a larger number of genotypes a rapid approach to processing the cores using core break count measurements was used. This technique was correlated to washed root length density measurements from core segments.

A tractor-mounted, hydraulic soil corer was used to drive sampling tubes into the soil to a depth of 2 m. The tubes were a molybdenum–steel alloy with an internal diameter of 42mm. The tubes are tipped with a head that is slightly swollen compared with the tube, but which tapers to slightly less than the internal diameter of the tube allowing the intact soil core to enter the tube with minimal disturbance. The intact soil cores were carefully removed onto a 2 m long cradle marked with 10cm increments. Each core was broken into 10cm segments from a depth of 20–200cm. At each depth the broken faces were observed and the number of exposed live roots counted (core-break counts; CBC). Roots were assessed as being from the current wheat crop based on colour (white) and the stiffness (live roots supple) (Watt *et al.*, 2008). It is important that the cores are broken with a snapping action rather than cut with a knife, as a cut will slice through the root leaving only a small cross section of root exposed which is very difficult to see. When broken, the roots are pulled from either of the faces of the break, and are easier to see. Hence, the counts on each face are later summed for each depth, as roots appearing on one face will not remain on the other.

### Correlation of core-break technique with root length density

To correlate between CBC and root length density, a subset of core segments were retained, bagged, and stored at 4 °C for root washing. Roots were separated from the retained core segments using a hydropneumatic elutriation system (Smucker *et al.*, 1982) with 0.078mm sieves. The washed root samples were stored in 50% ethanol. These samples were then scanned on a document scanner and processed with WinRHIZO® software. The batch scanning process used automatic grey scale thresholding, filtering out objects smaller than 0.1cm^2^ and with a length:width ratio <10.

**Fig. 3.**
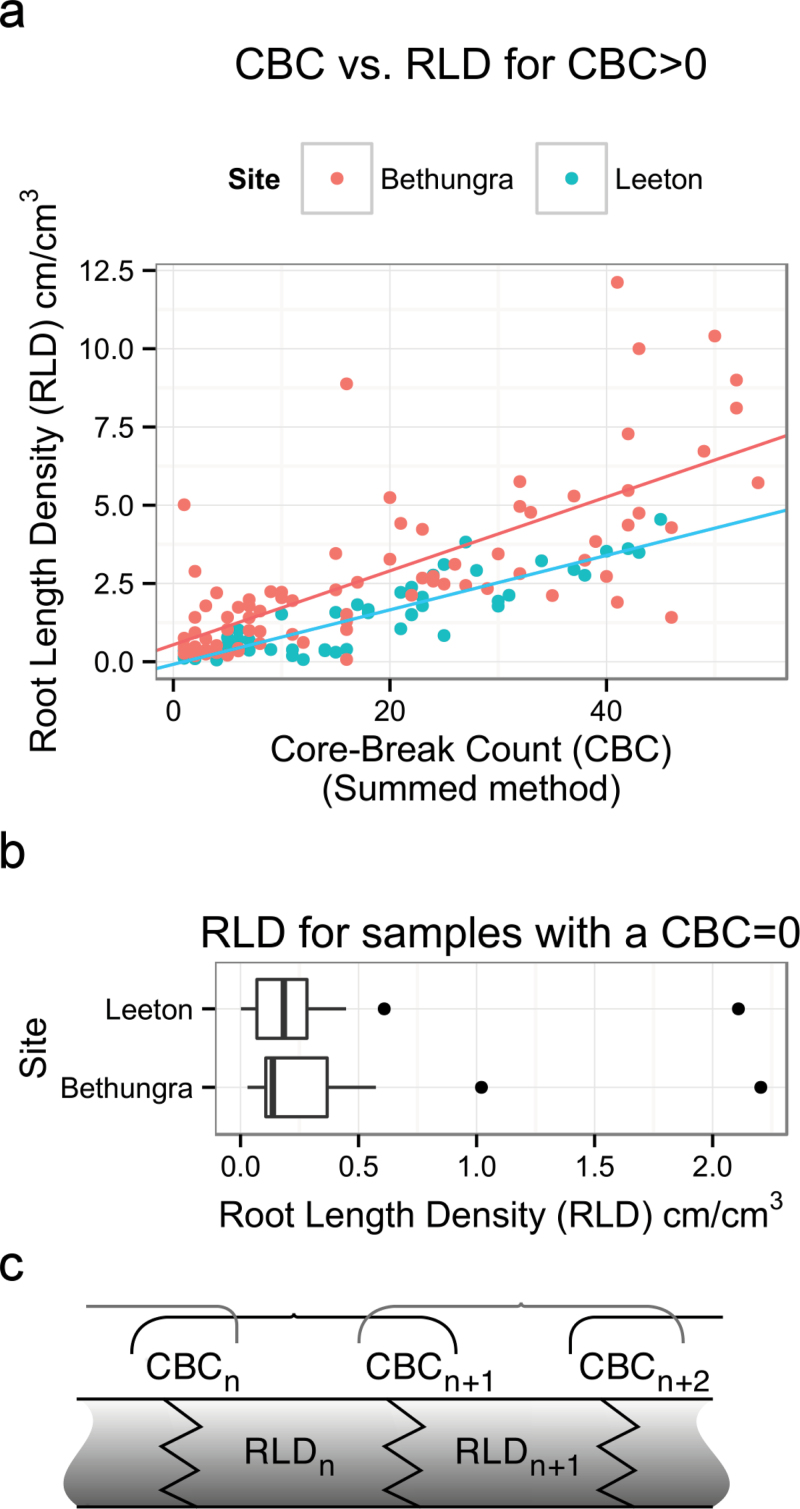
The relationship between CBC and RLD for Leeton and Bethungra in 2011. (a) A plot of CBC vs. RLD where the CBC>0. The lines are fitted regressions to the two data sets. The fit for Leeton was better than the fit for Bethungra. (b) A box plot representation of the range of RLDs for samples where the CBC=0. (c) A cartoon showing the ‘summed’ method for calculating CBC and correlating it with RLD: the sum of the CBC at depth *n* and the CBC at depth *n*+1 is correlated with the RLD for segment n: (corr(CBCn + CBCn+1, RLDn). (This figure is available in colour at *JXB* online.)

CBCs were converted to root-length densities (RLD), in cm cm^–3^, empirically, rather than using an existing modelled relationship (for example Grabarnik *et al.*, 1998). The CBCs and RLD for these samples were then correlated (Table 3).Three methods of processing the CBC data were assessed, as illustrated in Fig. 3c: (i) correlating the CBC at depth *n* with the RLD for segment *n* (corr(CBC*n*,RLD*n*), (ii) correlating the CBC at depth *n+1* with the RLD for segment *n* (corr(CBC*n+1*,RLD*n*), (iii) correlating the sum of the CBC at depth *n* and the CBC at depth *n+1* with the RLD for segment *n* (corr(CBC*n +* CBC*n+1*, RLD*n*). The third approach, the ‘summed method’, was chosen for the datasets because it gave a superior correlation at Leeton (*r* ^2^ of 0.80 vs. 0.73), although it was inferior correlation at Bethungra (*r* ^2^ of 0.53 vs. 0.66)

**Table 3.** Model and correlation of root length density (RLD) and core break count (CBC) where CBC>0 at Leeton and Bethungra (2011)

Site	Linear model	Coefficient of determination (r^2^)	*P*-value	Number of observations
Leeton	RLD=0.087∙CBC–0.084	0.80	<0.001	54
Bethungra	RLD=0.118∙CBC+0.543	0.53	<0.001	83

We chose not to include the CBC_0_ in the CBC ≈ RLD linear model because we did not intend to convert CBC_0_ to the intercept of the model, which would falsely suggest that roots were present through the length of the core. Instead when converting CBC for RLD for the purposes of comparisons between sites (Fig. 6) we chose to convert values where CBC=0 to an RLD of 0 with an uncertainty equal to the mean RLD_CBC=0_ plus the standard error of the RLD_CBC=0_. When converting the CBC>0 to RLD we propagated the uncertainty of the prediction from the regression generated linear model and combined it with the uncertainty from the repeated sampling to generate a combined standard error (Taylor, 1997).

**Table 4.** Plot measurements and calculations

Phenotype	Unit	Abbreviation	Calculation
Maximum depth	cm	MD	Calculated as the deepest layer (measured every 10cm) in which a root was detected.
Root penetration rate	cm/°C d	RPR	Calculated as the maximum depth over the time to flowering in growing degree days (°C days): $\frac{Maximum\;depth}{Flowering\;time}$
Total root length	cm core^–1^	TRL	Calculated by converting the CBCs to RLD at each depth, using the correlation established from the washed root samples, and taking the sum of the RLDs in the entire core
Shallow root length	cm core^–1^	SRL	Calculated as for TRL, but only to a depth of 60cm.
Deep root length	cm core^–1^	DRL	Calculated as for TRL, but only at depths greater than 130cm.
Deep to total root ratio.			Calculated as the ratio of deep root length to total root length: $\frac{Deep\;root\;length}{Total\;root\;length}$
Flowering time	°C day		The time from sowing to flowering as determined on the Zadoks decimal scale for wheat development (Z65). The time is thermal time in growing degree days (°C day) calculated using a base temperature of 0° C and using average daily temperature from meteorological records.
Hill plot biomass	g		The mass of the entire harvested hill plot.
Hill plot yield	g		The mass of the grain derived from the harvested hill plot.
100-grain Weight	g		The mass of 100-grains from the harvested hill plot.
Harvest index			$\frac{Hill\;plot\;yield}{Hill\;plot\;biomass}$
Green leaf score			The green leaf area of the penultimate leaf, flag leaf and head was scored on a weekly basis, post-flowering. These scores were integrated per unit of thermal time (°C day) after flowering for each plot until maturity.
Canopy temperature depression			The difference between the canopy temperature and the ambient temperature was measured a weekly basis, post- flowering. These measurements were integrated per unit of thermal time (°C day) after flowering for each plot until maturity.
Chlorophyll reflectance scores			The chlorophyll reflectance was measured on a weekly basis, post-flowering. These measurements were integrated per unit of thermal time (°C day) after flowering for each plot until maturity

### Shoot measurements

Three indirect shoot measures of a deep root system were also assessed (Fig. 2): green leaf (‘stay-green’) scores, chlorophyll reflectance and canopy temperature measurements. These were chosen as it was assumed that a root system accessing deep soil water would result in a longer green leaf area duration, more open stomates and lower leaf temperature. The relationship between plant performance and root traits was also measured to determine if the sowing configuration had created selective pressure for our desired root traits.

The shoot measures were normalized against the developmental speed of the genotypes, measured as the growing degree days to flowering. The Zadoks decimal system (Zadoks *et al.*, 1974; Bertholdsson, 1998) for categorizing the growth stages of wheat was scored on four replicated plots between the emergence of the head (Z49) and the end of flowering (Z70) on a weekly basis so that the time of flowering (Z65) could be accurately determined for each plot. Rainfall and temperature data were obtained from nearby Bureau of Meteorology stations. These records were used to calculate thermal time in growing degree days (°C day), which integrates the daily temperature with the number of days of growth, allowing for comparisons of growth rates between sites and seasons. Growing degree days are calculated for each day in the growing season by taking the average of the maximum temperature and the minimum temperature (or 0° C, whichever is higher).

Canopy temperature was measured with an infrared thermometer on a weekly basis post-flowering, weather permitting, and made in accordance with an established protocol (Rebetzke *et al.*, 2013). Briefly, measurements were made between 12.00 and 14.00h, with the sun behind the observer (on a fine, still, clear day). The thermometer was carefully aimed at exposed flag leaves but with procedure modified to account for hill plots not having complete canopies.

The amount of green leaf area was scored (hereafter ‘green leaf score’) on a weekly basis, post-flowering. A score of 1 was given for each of a completely green head, flag leaf, and penultimate leaf, for a maximum value of 3. The scores were then reduced as each of these organs senesced; e.g. a half-senesced flag leaf would receive a score of 0.5. The measurements were performed until no chlorophyll was observed and the hill had matured. Chlorophyll measurements were also performed between flowering and the time that the grain had reached maturity with a CM1000 Fieldscout®, given sufficient sunlight (measured by the instruments light meter). The instrument was aimed at the flag leaves of the hill plot.

### Phenotypes

Root and shoot phenotypes are summarized in Table 4. The maximum root depth (MD) was calculated as the deepest layer (measured every 10cm) in which a root was detected. ‘Root penetration rate’ (RPR) was calculated by dividing the maximum depth by the time to flowering in growing degree days (°C days) (Robertson *et al.*, 1993b; Kirkegaard and Lilley, 2007). Growing degree days were used to account for differences in climate between field environments and years. Some studies show downward root system extension ceases just after flowering (Troughton, 1962; see Gregory *et al.*, 1978; Thorup-Kristensen *et al.*, 2009; Kirkegaard *et al.*, 2007) although others show an increase in root length to maturity (Ford *et al.*, 2006; Manschadi *et al.* 2006). Total root length (TRL) was calculated by converting the CBCs to RLD at each depth, using the correlation established from the washed root samples, and taking the sum of the RLDs in the entire core. Alternatively, the RLDs were divided into two subsets, ‘shallow root length’ (SRL=<0.6 m) and ‘deep root length’ (DRL=>1.3 m).

Green leaf score and chlorophyll reflectance were calculated per °C day after flowering for each plot, representing the integrated amount and duration of green leaf. Canopy temperature depression (°C) was calculated per °C day after flowering for each plot, representing the integrated amount and duration of canopy temperature depression.

### Statistical analysis

Data was collated and plotted in the R environment using the ‘ggplot2’, ‘reshape2’, and ‘plyr’ packages. Mixed linear models were fitted with GenStat (16^th^ Edition, VSNi Ltd. UK), and planned comparisons at Bethungra and Leeton sites used run-range spatial models (Cullis *et al.* 1996). Run (being the row into which hill plots were sown) and range (being the order of hill plots in the row) were treated as random factors and genotype as a fixed factor in the initial analysis of genotype means. The fitted means and variance-covariance matrix were exported to R for *a priori* orthogonal contrasts for specific comparisons of interest. The site means used a mixed model. Site × run and site × range were treated as random factors and site and genotype as fixed factors. Correlations were calculated in GenStat. Unless otherwise stated statistical significance was at *P*=0.05.

## Results

### General observations of wheat growth in field environments

Above average in-season rainfall was experienced at both Bethungra and Leeton in 2011, although Leeton experienced a terminal drought towards the end of the grain filling period. Leeton experienced a similar season in 2010 and a drought in 2009. Bethungra experienced more rainfall than Leeton (770mm vs. 591mm); with longer growth seasons and more biomass and yield generated.

### Root phenotypes

**Fig. 4.**
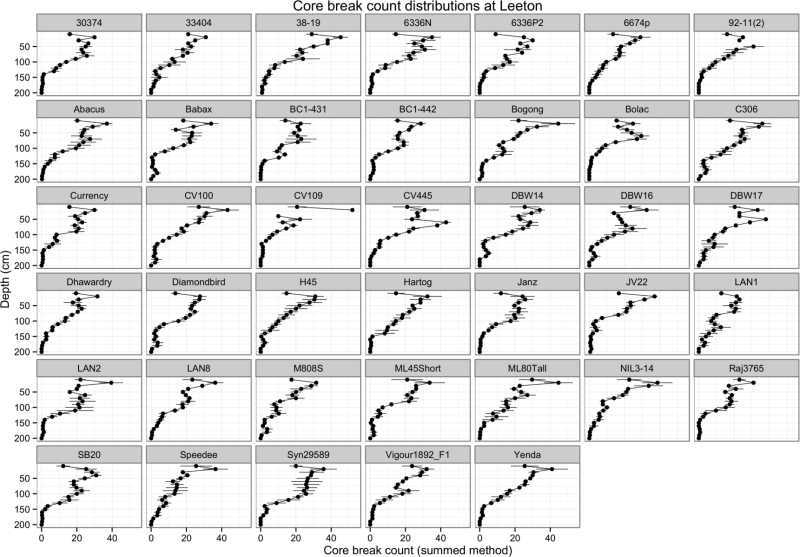
Root distributions by depth at Leeton Experimental Station 2011. The error bars show the standard error of the mean for four replicated observations. A similar plot of the data from Bethungra 2011 is in Supplemental Figure 1.

*Core break counts*: The root phenotype dataset was generated from core break counts (CBCs). The mean CBCs for the cored genotypes are presented in Fig. 4 for Leeton and in Supplemental Figure 1 for Bethungra. There seems to be variation in the ‘patterns’ of CBC distribution by depth for the different genotypes. However, the large error bars indicate substantial plot-to-plot variation. These CBC distributions cannot be compared between sites because the site relationships between CBC and RLD are different (below); only RLD distributions can be compared.

**Fig. 5.**
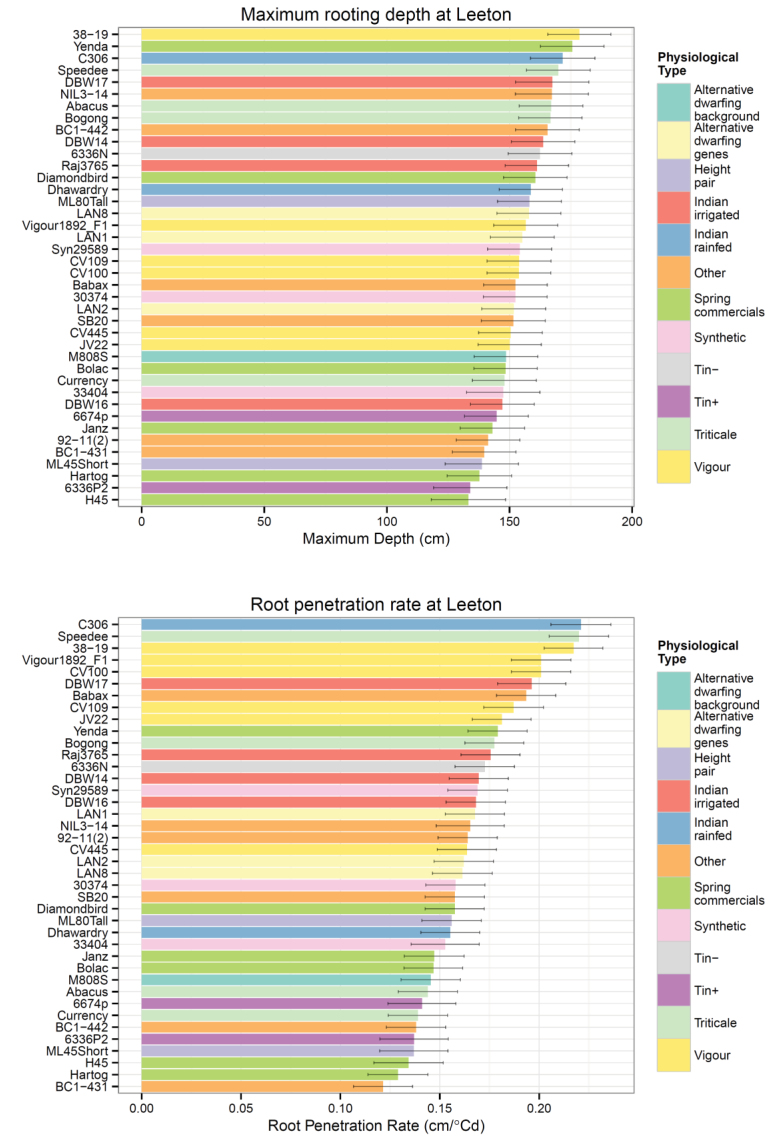
Maximum rooting depth and root penetration rate at Leeton in 2011. The data presented are predicted means and standard errors for a spatial model of the trial, which treated run and range as random factors.

Plotting the distributions of counts (Fig. 4 and Supplemental Fig. 1) can be used to gain a sense of which genotypes are partitioning their roots to depth. Interesting candidates include C306, Syn29589, DBW14, Speedee, and Babax. Other genotypes, such as H45 and ML45Short, appear to have RLD at depth, but the uncertainty encompasses zero. This highlights the way the data is constructed, as the mean and standard error of counts at each depth across four replicated cores. The result is that a genotype such as M808S, which seems to have deep roots in the distribution graph, is shown as having below average MD in Fig. 5 (below), where MD was calculated in each core and then adjusted by spatial modelling to give a genotype mean.

Random subsets of the collected cores were washed and the relationship between CBC and RLD calculated for both field environments. The relationships between the CBCs and RLDs where the CBC>0 for Bethungra and Leeton are shown in Figure 3(a). The linear model for Leeton was RLD=0.087 ∙ CBC–0.084 (*r* ^2^=0.80) and for Bethungra was RLD=0.118 ∙ CBC+0.543 (*r* ^2^=0.53, Table 3). The RLD of samples when the CBC=0 for both sites are presented in Fig. 3b, and can be used to estimate the sensitivity of the technique. The median RLD for Leeton is 0.18cm cm^–3^ and the upper quartile is 0.28cm cm^–3^. The median RLD for Bethungra is 0.13cm cm^–3^ and the upper quartile is 0.36cm cm^–3^. There were two outlier values at each site, between 0.5 and 2.5cm cm^–3^, which may be the result of root proliferation in a crack or pore between the break planes.

*Variation due to field environment*: The CBC distributions were used to generate summary statistics, such as maximum depth, and the significance of site and genotype on these parameters were analysed as factors in a mixed linear model (Table 5). The model took into account the spatial variation at each site. Site was a significant factor for maximum depth, root penetration rate, and shallow root length. Site was not significant for deep root length, although it was significant for the ratio of deep root length to total root length. Maximum depth, root penetration rate, and the ratio of deep to total roots were greater at Leeton, whereas total root length and shallow root length were greater at Bethungra (Table 5). Deep root length was not distinguishable between the two sites, 1.2±0.6 vs. 0.7±0.2cm per core.

**Table 5.** Statistical significance of site and genotype as factors influencing root traits The data was for lines grown at Leeton and Bethungra in 2011. Traits were modelled as dependent variables in a mixed linear model with site and genotype as fixed factors and site×row and site×column as random factors to account for spatial variability. Least squares means±standard errors were obtained from the full model.

Trait	Significance		Least squares means	
	Site	Genotype	Bethungra	Leeton
Root penetration rate (cm/°C d)	<0.001^***^	<0.001^***^	0.119±0.005	0.167±0.003
Maximum depth (cm)	0.003^**^	0.755	139.7±4.3	155.6±3.0
Total root length (cm per core)	0.767	0.249	27.6±3.4	18.6±1.8
Shallow root length (cm per core (Depth 20–60cm))	<0.001^***^	0.188	18.4±1.9	9.4±0.9
Deep root length (cm/core (Depth>130cm))	0.292	0.909	1.2±0.6	0.7±0.2
Deep to total root ratio.	<0.001^***^	<0.001^***^	0.015±0.007	0.037±0.005
Flowering time (°C day)	<0.001^***^	<0.001^***^	1161±7	946±4
Hill plot biomass (g)	<0.001^***^	<0.001^***^	292.9±6.7	175.9±6.6
Hill plot yield (g)	<0.001^***^	<0.001^***^	106.54±2.26	73.50±2.24
100-grain weight (g)	0.496	<0.001^***^	3.559±0.040	3.590±0.039
Harvest index	<0.001^***^	<0.001^***^	0.362±0.003	0.417±0.003

*P*-values are given as: 0 ‘***’ 0.001 ‘**’ 0.01 ‘*’ 0.05 ‘.’ 0.1 ‘ ‘ 1

*Variation due to genotype*: Genotype was a significant factor for the root penetration rate and the ratio of deep to total root length, but it was not significant for maximum depth, total root length, shallow root length, or deep root length. This reflects the very high level of plot-to-plot variation for these factors.

Planned comparisons revealed few differences in root traits based on genotypes selected for physiological types suspected to generate contrasting root growth (Table 6). The only comparison that was significantly different for maximum depth was between Indian-derived varieties selected for rainfed and irrigated conditions. The rainfed varieties had significantly higher maximum depth and root penetration rate but only at Bethungra. The lack of significance for other comparisons may reflect the high level of plot-to-plot variation for maximum depth.

**Table 6.** Planned comparisons of selected wheat genotypes sown and cored at Leeton and Bethungra in 2011

Comparison	Maximum depth				Root penetration rate			
	Leeton		Bethungra		Leeton		Bethungra	
	Result	*P*-value	Result	*P*-value	Result	*P*-value	Result	*P*-value
Early vigour vs. spring commercials	n.s.	0.31	n.s.	0.57	Vigour > commercials	3.8 e–07^***^	n.s.	0.53
Triticales vs. spring commercials	n.s.	0.10			Triticales > commercials	0.02^**^		
Synthetic wheats vs. spring commercials	n.s	0.86	n.s.	0.38	n.s.	0.30	n.s.	0.39
Tiller inhibition + vs. wild type.	n.s.	0.14	n.s.	0.68	n.s.	0.11	n.s.	0.70
Rainfed vs. irrigated wheats	n.s.	0.64	Rainfed > irrigated wheats	0.009^***^	n.s.	0.39	Rainfed > irrigated wheats	0.01^**^
Short vs. tall	n.s.	0.31			n.s.	0.39		
SB20 vs. Hartog	n.s.	0.44			n.s.	0.16		
SB20 vs. Babax	n.s.	0.96			n.s.	0.07		
Babax vs. Hartog	n.s.	0.41			Babax > hartog	1.6e–03^***^		
Alternative dwarfing genes vs. background	n.s.	0.66	n.s.	0.30	n.s.	0.27	n.s.	0.29

P-values are given as: 0 ‘***’ 0.001 ‘**’ 0.01 ‘*’ 0.05 ‘.’ 0.1 ‘ ‘ 1

Lines selected for greater early vigour, the triticales and synthetic wheats were contrasted with commercial spring wheats on the presumption of high levels of root growth vigour. Early vigour genotypes had significantly greater root penetration rate at Leeton, but not at Bethungra. Triticales also had significantly greater root penetration rate at Leeton, but were not sampled at Bethungra. Synthetic wheats were not significantly different to standard commercial wheats.

Other comparisons were between near-isogenic-lines (NILs), genotypes differing only for the *tin* gene or alternative dwarfing genes, none of which showed significant differences for maximum depth or root penetration rate at either site. SB20 was a RIL derived from a cross of Seri, a moderately drought-tolerant semi-dwarf wheat, and Babax, a strongly drought tolerant semi-dwarf wheat (Olivares-Villegas *et al.*, 2007). SeriM82 and Hartog have previously been compared in a rhizobox study with SeriM82 and shown to have significantly greater root density at depth (Manschadi and Christopher, 2006). Babax, Hartog and SB20 were compared at Leeton. Babax had a faster root penetration rate than Hartog, but other comparisons were not significant.

*Post-hoc* comparisons of maximum depth and root penetration rate compared the best and worst genotypes at Leeton and Bethungra (five and four genotypes, respectively) and the commercial wheats (Table 7). Only three of the five deepest genotypes were the fastest growing, whereas in the smaller population at Bethungra the four genotypes were the same in both categories. The genotype C306, grown at both sites, was in the best grouping for all four categories. The genotype CV100 was represented amongst the best except in the category of maximum depth at Leeton.

**Table 7.** Post-hoc *testing of selected wheat genotypes sown and cored at Leeton and Bethungra in 2011*

*Post-hoc* testing				
	Leeton		Bethungra	
Root penetration rate.				
Highs	C306, Speedee, 38-19, Vigour1892_F1, CV100		C306, CV100, LAN8, NIL3-14	
Lows	6336P2, ML45Short, H45, Hartog, BC1-431		38-19, LAN4, Drysdale, DBW14	
Spring commercials	H45, Bolac, Diamondbird, Hartog, Janz, Yenda		Drysdale, Yenda	
Highs vs. lows	Highs > lows	4.12e–18^***^	Highs > lows	8.66e–06^***^
Highs vs. spring commercials	Highs > commercials	5.61e–13^***^	Highs > commercials	0.02^**^
Lows vs. spring commercials	n.s.	0.12	n.s.	0.19
Maximum depth				
Highs	38-19, Yenda, C306, Speedee, DBW17		C306, CV100, LAN8, NIL3-14	
Lows	BC1-431, ML45Short, Hartog, 6336P2, H45		38-19, LAN4, Drysdale, DBW14	
Spring commercials	H45, Bolac, Diamondbird, Hartog, Janz, Yenda		Drysdale, Yenda	
Highs vs. lows	Highs>lows	1.53e–05^***^	Highs>lows	7.36e–06^***^
Highs vs. spring commercials	Highs > commercials	0.01^**^	Highs > commercials	0.02^**^
Lows vs. spring commercials	n.s.	0.20	n.s.	0.17

P-values are given as: 0 ‘***’ 0.001 ‘**’ 0.01 ‘*’ 0.05 ‘.’ 0.1 ‘ ‘ 1

**Fig. 6.**
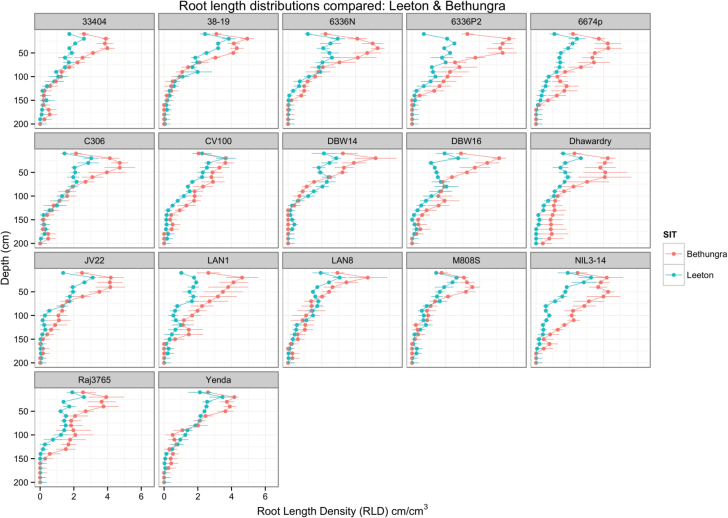
Root distributions for Leeton and Bethungra in 2011. Root length density (RLD) is expressed in cm per cm^3^. RLD was calculated using the relationship to core break counts (CBC) in Table 4. The error bars show the standard error of the mean for four replicated observations. (This figure is available in colour at *JXB* online.)

Between Leeton and Bethungra, 17 genotypes were compared in 2011, and their root distributions are shown in Fig. 6. Some entries, such as 38-19, Raj3765, M808S, and LAN1 rooted more deeply at Leeton than Bethungra, whereas the opposite seemed true only for variety Dharwar dry at Bethungra. This accords with the results of the mixed model in Table 5, which shows least squares means for maximum depth and root penetration rate at Leeton of 155.6±3.0cm and 0.167±0.003cm/°C d, respectively, contrasting with maximum depth and root penetration rate at Bethungra of 139.7±4.3cm and 0.119±0.005cm/°C d, respectively. The mean total root length at Bethungra was larger than at Leeton, 27.6±3.4cm/core compared with 18.6±1.8cm/core. Interestingly, most of this increase would be attributed to differences in the shallow root length, between 20 and 60cm in depth, and not in the deep root length, below 130cm in depth, which did not vary for site (Table 5).

**Fig. 7.**
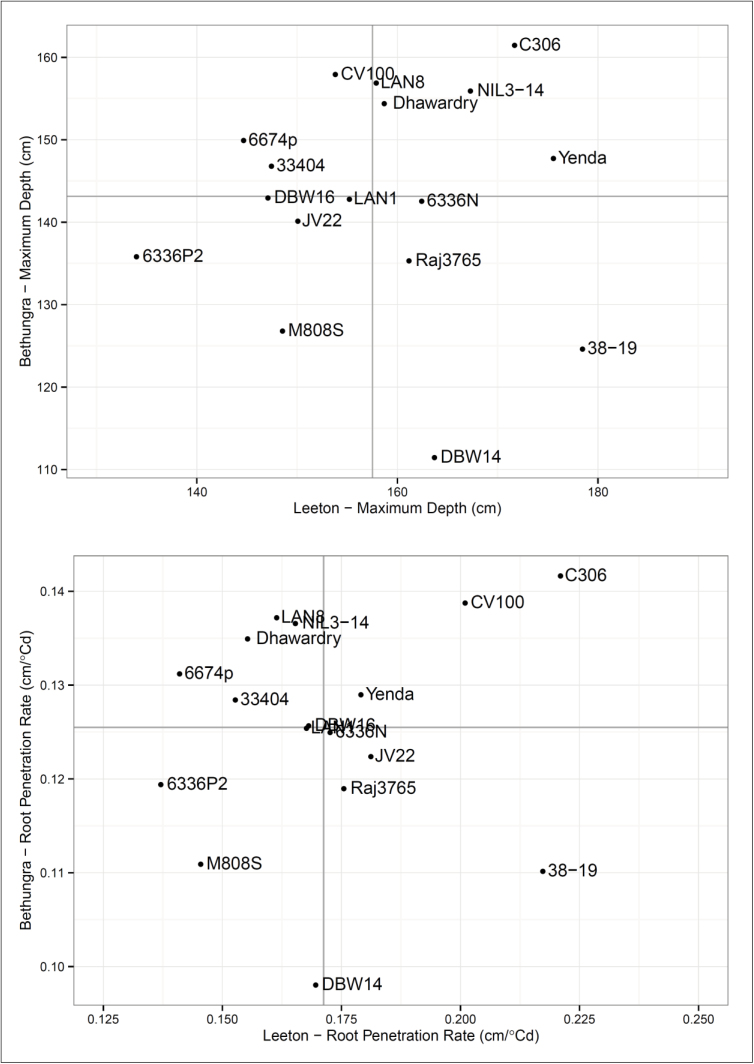
MD and RPR at Leeton and Bethungra in 2011.

Figure 7 shows the comparative performance at each site. The genotypes in the upper right quadrant, such as C306, had above average performance at both sites; whereas genotypes in the lower left quadrant, such as 6336P2 and M808S, had lower than average performance at both sites. The performance of genotypes in the remaining two quadrants indicates potential interaction of genotype and environment for maximum depth and root penetration rate. For example, the genotype CV100 had above average performance at Bethungra, but average performance at Leeton.

**Fig. 8.**
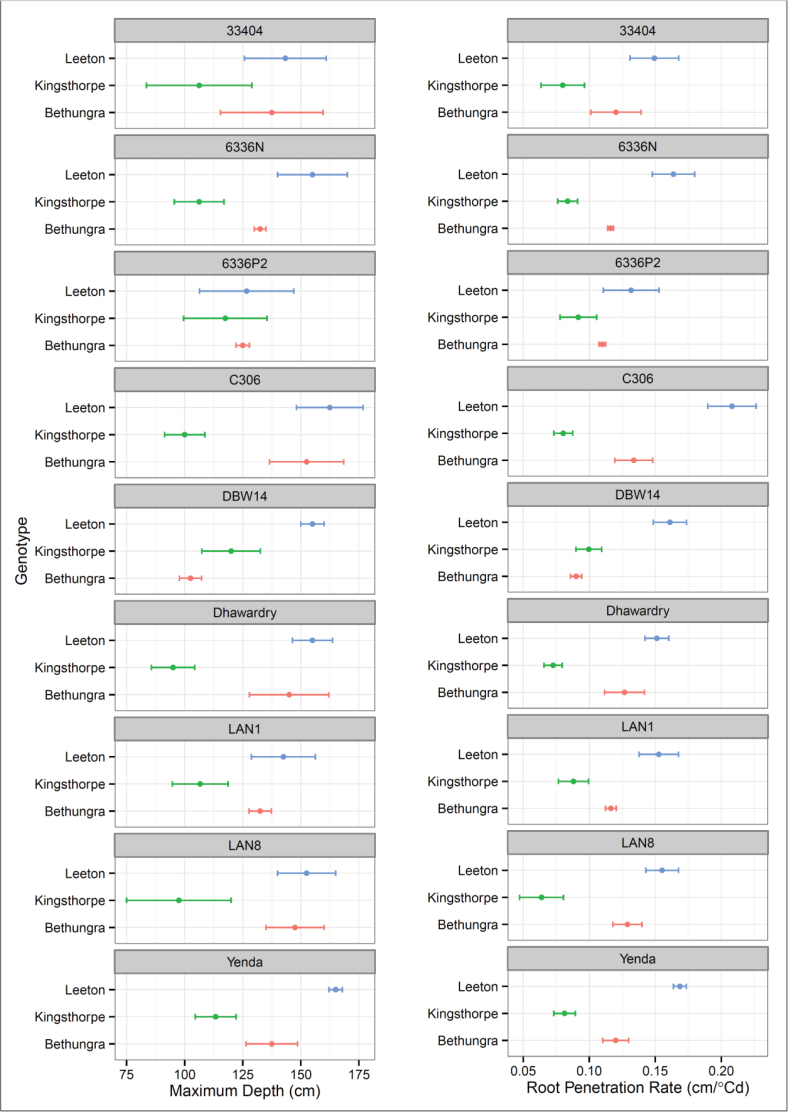
Max depth at Leeton, Bethungra, and Gatton 2011. Error bars are the standard error of the mean with four observations. (This figure is available in colour at *JXB* online.)

Differences in the maximum depth of genotypes sown at Leeton, Bethungra and Kingsthorpe in 2011 are shown in Fig. 8. Some genotypes such as Yenda, DBW14, and 6336N performed differently across all three sites whereas others, such as 33404 and the tin-containing 6336P2, maintained their high ranking across all sites.

**Fig. 9.**
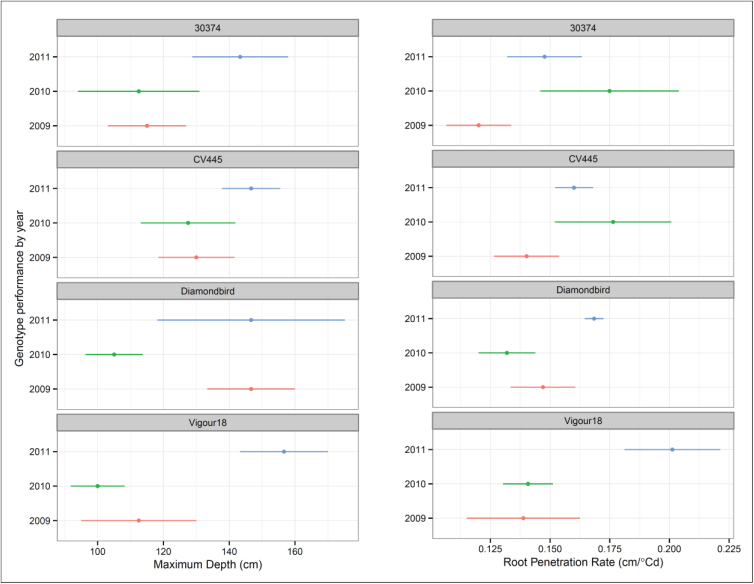
Maximum depth and root penetration rate at Leeton in 2009, 2010, and 2011. (This figure is available in colour at *JXB* online.)

The maximum depth for four genotypes grown in Leeton in 2009, 2010, and 2011 are shown in Fig. 9. The entries 30374 and CV445 did not differ between the three seasons. Diamondbird rooted shallower in 2010, a wet year, than the drier 2009 and 2011. Vigour 18 was deeper in the driest year, 2011, than in the preceding two.

The value of selections from physiological types is reflected in the *post-hoc* comparisons in Table 7. The five best genotypes for maximum depth and root penetration rate at Leeton, and the four best genotypes at Bethungra, were significantly better than the five and four worst genotypes, respectively. Importantly, the best genotypes at both sites were also significantly better than the commercial spring wheats whereas the worst genotypes were not significantly poorer.

### The links between indirect shoot and direct root phenotypes

Table 8 shows correlations of various root parameters with four performance parameters: grain yield, total biomass, harvest index, and 100-grain weight. The strongest correlation was –0.29 between harvest index and ratio of deep to total root length. Green leaf scores, CTD measurements, and chlorophyll reflectance measurements were taken at regular intervals after flowering. These measurements approach zero as the plot matured and are therefore related to flowering time. To account for this relationship, the area under the curve between flowering and maturity was integrated for both traits in an attempt to account for flowering time differences between genotypes. There was a weak but significant correlation between maximum depth and green leaf score (–0.5), CTD (0.45), and chlorophyll reflectance measurements (0.32). This was the only root measure to show a correlation with these shoot measures.

**Table 8.** Correlation table of yield and root parameters for material grown at Leeton in 2011 Traits were modelled as dependent variables in a mixed model with genotype and site as fixed factors and site×run and site×range as random factors to account for spatial variability. Least squares means and standard errors were generated from the model.

	Root penetration rate (cm/°c d)	Maximum depth (cm)	Total root length (cm/core)	Shallow root length (cm/ core (depth 20–60cm))	Deep root length (cm/core (depth>130cm))	Deep to total root length ratio
Hill plot yield (g)	0.04	–0.08	0.12	–0.11	0.06	0.01
Hill plot biomass (g)	0.16	0.01	0.12	–0.1	0.2	0.17
Harvest index	–0.27	–0.16	–0.03	–0.01	–0.26	–0.29*
100-grain weight (g)	0.02	0.13	0.22	0.08	0.28	0.22
Green leaf score	–0.12	–0.5***	–0.01	0.09	–0.17	–0.2
Canopy temperature depression	0.09	0.45**	0.06	0.02	0.21	0.21
Chlorophyll reflectance scores	0.07	0.32*	–0.01	–0.05	0.2	0.2

P-values were: 0 ‘***’ 0.001 ‘**’ 0.01 ‘*’ 0.05 ‘.’ 0.1 ‘ ‘ 1.

## Discussion

This study sought to identify wheat germplasm containing novel deep root traits with potential for integration into breeding programmes using direct root phenotyping approaches in field environments, and to assess these direct approaches against indirect shoot phenotypes. Four traits were proposed for an ideotypic wheat for rain-fed production where deep water is important (Wasson *et al.*, 2012): (i) deeper roots; (ii) increased root length density in medium and deep soil layers; (iii) reduced root length density in the topsoil; and (iv) increased root hair growth and xylem diameter. The first three of these traits were assessed in this study. We found that soil coring at grain development revealed variation in these, owing to environment and to genotype. Shoot phenotypes were not reliably related to the deep root phenotypes.

### The core break method for phenotyping roots

The core break counts were the central measurements for phenotyping roots. Although rapid (100–200 cores per day) there are some limitations to this method. The high root density in the top 10cm of soil around the crowns was difficult to assess accurately and rapidly with core break counts, and correlated poorly with RLD from washed and scanned root samples. Hence we made no attempt to quantify topsoil root length density from our data. Assessing topsoil RLD in the field might be better accomplished with a qualitative or semi-quantitative estimate. A ‘shovelomics’ approach using semi-quantitative strategy has been used in maize to assess root number, type, and angle in the field (Trachsel *et al.*, 2010). Riley *et al.* recently used DNA levels in surface soils to assess root activity (2010).

The correlation between CBC and RLD was better at Leeton than Bethungra. The core break method assumes that roots are randomly distributed in three dimensions, which is unlikely in structured soil (White and Kirkegaard 2010). Because the soil at Bethungra is denser (higher bulk density) it may force the roots to exploit more cracks and pores, reducing the random nature of the root distribution (White and Kirkegaard 2010). An additional source of variation may be the lighter colour of the Bethungra soil, which has a poor contrast with the light coloured roots making accurate counting more difficult.

The relationship between CBC and RLD presents difficulties in the interpretation and presentation of the data where CBC=0. The RLDs for samples where the CBC=0, represented in Fig. 3b show that very few samples at Leeton, and no samples at Bethungra, had zero RLD (although the median RLDs for these samples were small). However, we do not believe that this indicates the presence of roots where CBC=0. Some of the small root lengths may result from experimental error, for example, carryover from the washing process using the hydropneumatic root elutriation system, but some will be lengths of root not detected in the core break. The upper quartiles of these RLDs were lower than 0.4cm cm^–3^, whereas physiologically relevant RLDs for water uptake are 0.1cm cm^–3^ in wheat and 0.2cm cm^–3^ in sorghum (Kirkegaard *et al.* 2007; Robertson *et al.*, 1993a). The ‘sensitivity’ of the technique is close to the small RLDs sufficient for water uptake, but should be improved upon to ensure these small but relevant lengths of root are detected.

### Shoot phenotypes as indicators of root phenotypes

Our attempts to identify genotypes with the deepest root traits using non-destructive indirect shoot measures were largely unsuccessful, and there was little correlation with hill plot parameters (Table 8). There were weak positive correlations between maximum depth and both CTD (0.45) and chlorophyll reflectance (0.32), and a weak negative correlation with green leaf score (–0.5). There were no significant correlations with other root traits.

Canopy temperature can be an indirect measure of plant water status, although it is not the only factor that may cause stomata to close (Rebetzke *et al.*, 2013). As a proxy for root traits others (e.g. Villegas *et al.*, 2000; Royo *et al.*, 2002; Lopes and Reynolds, 2010) reported that wheat genotypes with more root growth at depth had cooler canopies, from more green leaf area and/or transpiration. The ‘canopy’ of a hill plot is structured differently, and although it can be measured using infrared thermometrics, the boundary layer between leaves and the circulating air in the hill plot canopy is likely to be somewhat different. The danger of boundary layer affecting CTD across genotypes was demonstrated in Rebetzke *et al.* (2013).

Like CTD, the stay-green phenotype is thought to indicate an enduringly high plant water status and hence a root system with access to water. There was a lack of correlation between root measures and either green leaf scores or chlorophyll reflectance. The stay-green phenotype seems to be more relevant to tolerance to intermittent drought rather than terminal drought and where there is a high probability of rainfall during grain filling (Ludlow and Muchow, 1990; Thomas and Smart, 1993; Thomas and Howarth, 2000; Mahalakshmi and Bidinger, 2002; Richards *et al.*, 2002). Our study found only very weak evidence linking green leaf score and root traits.

### Field environment and root phenotypes

Figure 6 shows the comparative root growth for genotypes grown at both Bethungra and Leeton in 2011. Site means for the traits (Table 5) show that the shallow (10–60cm) RLD at Bethungra was significantly larger, whereas maximum depth and root penetration rate at Leeton were greater. Root penetration rates for the Bethungra soil type are similar to values reported elsewhere (1.3cm/°C d, Kirkegaard and Lilley, 2007). Most genotypes grew more roots at shallow depths in Bethungra and genotypes grew deeper roots at Leeton. The Bethungra soil is denser than the Leeton soil with more plant available water in the shallow layers (0–60cm, 97.9 vs. 84.6mm), but similar amounts in the deeper layers (60–180cm, 104.1 vs. 106.2mm, Table 2). Deep root growth may also be favoured in Leeton because the density of the soil was lower. Furthermore, Bethungra received more in-season rainfall than Leeton (770 vs. 591mm). The drier surface soil at Leeton may have limited the production of shallow roots. Blum and Ritchie (1984) showed that a dry topsoil limited crown root growth and caused photoassimilate to be redirected to the primary roots which grew deeper into the soil. A similar result was obtained by Asseng *et al.* (1998) in wheat.

The genotype C306 stood out for its performance at both sites, both in terms of root penetration rate and maximum depth (Fig. 7). C306 is an Indian variety released in 1965 (Subbiah *et al.*, 1968). It is a tall wheat (110–120cm) with a medium developmental time (136–140 days) suitable for rainfed conditions in India’s North Eastern and North Western Plains Zones (from Rajasthan to Nagaland and including Orissa). At both Leeton and Bethungra the variety seems to have an extensive shallow root system as well as roots at depth. The genotype CV100 also exhibited fast root penetration rate at both sites; however this only translated to a large maximum depth at Bethungra. At Leeton the genotype flowered and matured rapidly, resulting in a below average maximum depth. A contrasting genotype was a spring wheat version of a Russian winter wheat background Mironovskaya (M808S), which had below average root penetration rate and maximum depth at both sites. Other genotypes demonstrate plastic responses. 38-19, a spring wheat genotype from a vigour breeding programme, exhibited root penetration rate and maximum depth that was above average at Leeton, but below average at Bethungra. The opposite was true for the genotypes 6674P, a spring wheat with the tiller inhibition gene, and 33404, a synthetic wheat.

These genotypes would be promising candidates for investigating the genetic and physiological drivers of root traits. Evidence of genotypes with stable root penetration rate in differing environments, such as C306 and M808S, suggest that maximum depth can be increased by combining high root penetration rate with long maturity characteristics, and that these characteristics are genetically separate. Other genotypes, such as 38-19 and 33404, provide evidence that the root penetration rate may have other drivers. It is unclear if root penetration rate is a function of the ability to explore the soil for pores or cracks, or to grow through hard soil; nor is it clear if these characteristics can be combined in a single root system.

The root penetration rate, defined here as the maximum depth over the thermal time to flowering, which is widely thought to be the point at which downward root growth ceases, was assessed here as an alternative trait to maximum depth. It was hoped that root penetration rate would vary less between season and site, and would be a better guide to the potential of a variety to generate deep root systems. Our study reveals that root penetration rate varied significantly with both genotype and site. Several factors might account for the site variability in the trait. Firstly, it is likely that many factors, in addition to flowering time, could influence the development rate, such as soil physical constraints and nutritional factors. Soil temperature is another potential constraint, but it should be partially accounted for by our measure of flowering time, which integrates growing temperatures. Secondly, root systems may not cease downward growth at flowering. Post-anthesis (flowering) root growth is a trait that could be considered as ideotypic for certain environments if the gain in accessing deeper soil water exceeded the cost of fuelling additional root growth.

### Genotype and root phenotypes

The genotype means for maximum depth and root penetration rate at Leeton and Bethungra in 2011 (Fig. 5 and Supplemental Fig. 2) and the planned comparisons of the different physiological types (Table 6) are based on the same spatially modelled data. The large standard errors associated with the genotype means indicate that real differences may be difficult to detect with an alpha of 0.05, and the findings of non-significance should be treated cautiously. These errors may be experimental, related to the technique, or they may be real responses driven by plasticity in the root development or root responses to microenvironments in the soil.

Indian rainfed genotypes grew deeper root systems than Indian irrigated varieties at Bethungra, but, perhaps surprisingly, the difference was not apparent at Leeton. Reflecting the maximum depth result, Indian rainfed wheats grew faster than Indian irrigated wheats at Bethungra but not at Leeton. Perhaps further consideration should be given to what root system traits allow wheat to best exploit irrigation, as they may not be radically different to rainfed traits, at least in some soil environments.

Three of the four triticales sown seemed to have grown deep root systems, but when compared with the commercial spring wheats the comparison was outside of the range of significance with a *P*-value of 0.1. Root penetration rate, which takes into account flowering time, showed that triticales grew faster than spring commercial wheats. Vigour wheats grew faster than spring commercials at Leeton, but not at Bethungra. Consideration should be given to whether high root penetration rate is genetically separate from flowering time, and whether it can be combined with a longer duration to deliver deeper root systems. However, the value of selections from physiological types may be better reflected in the *post-hoc* comparisons in Table 7. The five best genotypes for maximum depth and root penetration rate at Leeton, and the four best genotypes at Bethungra, were significantly better than the five and four worst genotypes, respectively. Importantly, although best genotypes at both sites were also significantly better than the commercial spring wheats, whereas the worst genotypes were not significantly poorer.

## Conclusions

The best genotypes for root traits in the study outperformed the poorest genotypes and the commercial varieties in a *post-hoc* analysis. Therefore, the field coring approach generally selected for material that, in terms of root parameters, are outperforming those current varieties provided by breeding programmes to farmers. This suggests that there may be merit to the approach of combining genotype selection using physiological background and direct measurement of root traits using soil coring. Indirect measurements were not generally good predictors of root traits in the hill plot sowing configuration, but the significant negative correlation between green leaf score and maximum depth could be explored in greater depth.

## Supplementary data

Supplementary data are available at *JXB* online

Figure S1. Root distributions by depth at Bethungra 2011. The error bars show the standard error of the mean for four replicated observations.

Figure S2. Maximum rooting depth and root penetration rate at Bethungra in 2011. The data presented are predicted means and standard errors for a spatial model of the trial, which treated run and range as random factors.

Supplementary Data
