# Supplementary material for: Soil coring at multiple field environments can directly quantify variation in deep root traits to select wheat genotypes for breeding
Source: J Exp Bot. 2014 Jun 24;65(21):6231–49. doi: 10.1093/jxb/eru250 (PMC4223987; doi:10.1093/jxb/eru250)
Supplement: Supplementary Data [file supp_65_21_6231__index.html]

Soil coring at multiple field environments can directly quantify variation in deep root traits to select wheat genotypes for breeding — Soil coring at multiple field environments can directly quantify variation in deep root traits to select wheat genotypes for breeding — Supplementary Data 

# Soil coring at multiple field environments can directly quantify variation in deep root traits to select wheat genotypes for breeding

## Supplementary Data

Data files

**Files in this Data Supplement:**

- Supplementary Data - Supplementary Data
- Supplementary Data - Supplementary Data
