# Supplementary material for: Soil coring at multiple field environments can directly quantify variation in deep root traits to select wheat genotypes for breeding
Source: J Exp Bot. 2014 Jun 24;65(21):6231–49. doi: 10.1093/jxb/eru250 (PMC4223987; doi:10.1093/jxb/eru250)
Supplement: Supplementary Data [file supp_eru250_Supplementary_Files.pdf]

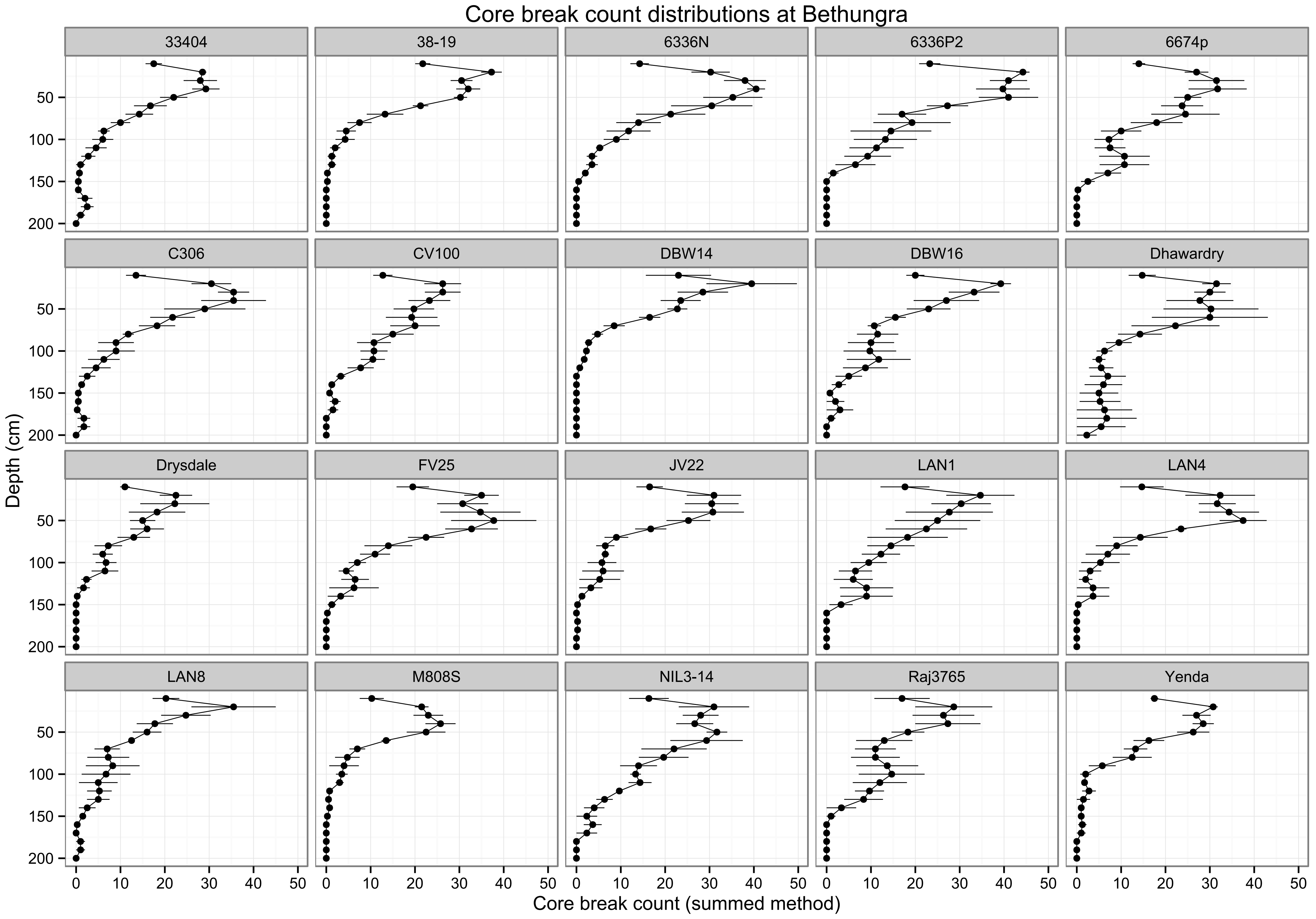

Core break count distributions at Bethungra
33404
38-19
6336N
6336P2
6674p
C306
CV100
DBW14
DBW16
Dhawardry
Drysdale
FV25
JV22
LAN1
LAN4
LAN8
M808S
NIL3-14
Raj3765
Yenda
Depth (cm)
Core break count (summed method)

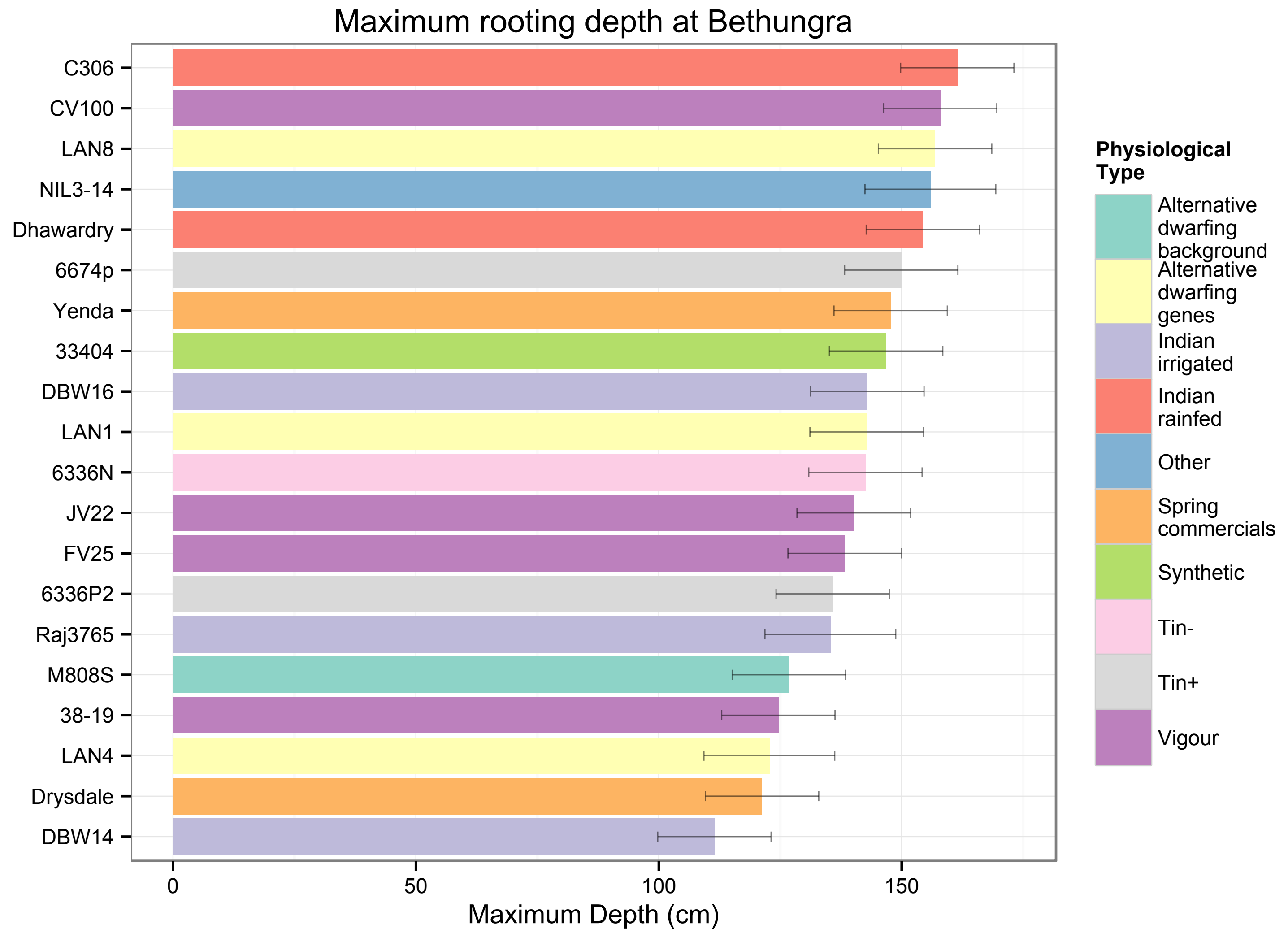
Maximum rooting depth at Bethungra
C306
CV100
LAN8
NIL3-14
Dhawardry
6674p
Yenda
33404
DBW16
LAN1
6336N
JV22
FV25
6336P2
Raj3765
M808S
38-19
LAN4
Drysdale
DBW14
0
50
100
150
Maximum Depth (cm)
Physiological Type
Alternative dwarfing background
Alternative dwarfing genes
Indian irrigated
Indian rainfed
Other
Spring commercials
Synthetic
Tin-
Tin+
Vigour

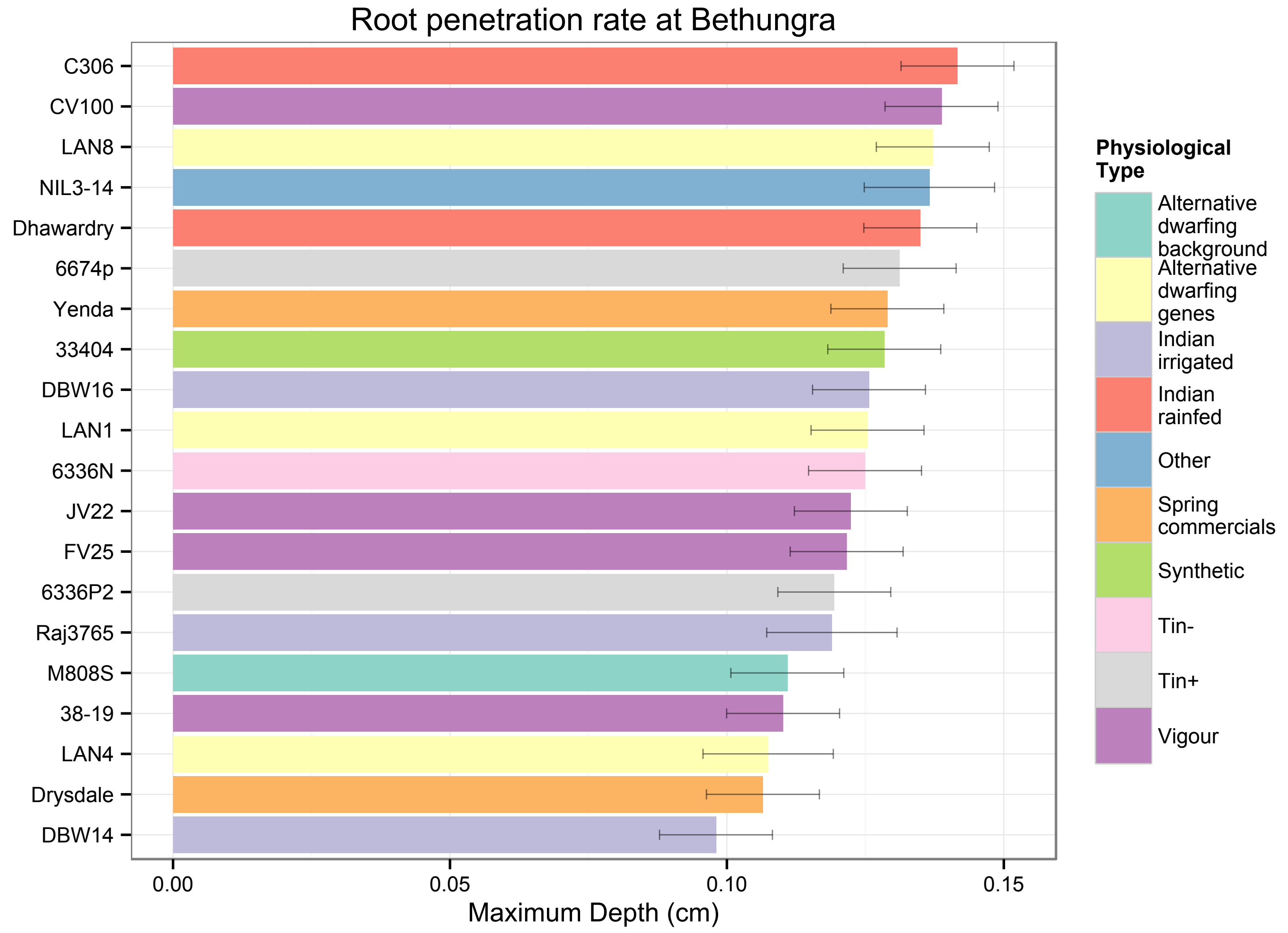
Root penetration rate at Bethungra
C306
CV100
LAN8
NIL3-14
Dhawardry
6674p
Yenda
33404
DBW16
LAN1
6336N
JV22
FV25
6336P2
Raj3765
M808S
38-19
LAN4
Drysdale
DBW14
0.00
0.05
0.10
0.15
Maximum Depth (cm)
Physiological Type
Alternative dwarfing background
Alternative dwarfing genes
Indian irrigated
Indian rainfed
Other
Spring commercials
Synthetic
Tin-
Tin+
Vigour
