# Supplementary material for: Soil coring at multiple field environments can directly quantify variation in deep root traits to select wheat genotypes for breeding
Source: J Exp Bot. 2014 Jun 24;65(21):6231–49. doi: 10.1093/jxb/eru250 (PMC4223987; doi:10.1093/jxb/eru250)
Supplement: Supplementary Data [file supp_eru250_Supplementary_Data.docx]

**Supplementary Data may be found at JXB online**

Supplemental Figure 1. Root distributions by depth at Bethungra 2011. The error bars show the standard error of the mean for four replicated observations.

Supplemental Figure 2. Maximum rooting depth and root penetration rate at Bethungra in 2011. The data presented are predicted means and standard errors for a spatial model of the trial, which treated run and range as random factors.
